# Identification and Characterization of Known Biallelic Mutations in the *IFT27* (*BBS19*) Gene in a Novel Family With Bardet-Biedl Syndrome

**DOI:** 10.3389/fgene.2019.00021

**Published:** 2019-01-30

**Authors:** Elise Schaefer, Clarisse Delvallée, Laura Mary, Corinne Stoetzel, Véronique Geoffroy, Caroline Marks-Delesalle, Muriel Holder-Espinasse, Jamal Ghoumid, Hélène Dollfus, Jean Muller

**Affiliations:** ^1^Laboratoire de Génétique Médicale, Institut de Génétique Médicale d’Alsace, INSERM U1112, Fédération de Médecine Translationnelle de Strasbourg, Université de Strasbourg, Strasbourg, France; ^2^Service de Génétique Médicale, Hôpitaux Universitaires de Strasbourg, Strasbourg, France; ^3^Laboratoire de Diagnostic Génétique, Hôpitaux Universitaires de Strasbourg, Strasbourg, France; ^4^Service d’Exploration de la Vision et Neuro-Ophtalmologie, Hôpital Roger Salengro, CHRU Lille, Lille, France; ^5^Clinical Genetics Department, Guy’s Hospital, London, United Kingdom; ^6^Service de Génétique Clinique, Hôpital Jeanne de Flandre, CHRU Lille, Lille, France

**Keywords:** Bardet-Biedl syndrome, *IFT27* gene, *BBS19*, ciliopathy, whole exome sequencing

## Abstract

Bardet-Biedl syndrome (BBS; MIM 209900) is a rare ciliopathy characterized by retinitis pigmentosa, postaxial polydactyly, obesity, hypogonadism, cognitive impairment and kidney dysfunction. Mutations in 22 BBS genes have been identified to cause the disease. We report a family with typical BBS features (retinitis pigmentosa, postaxial polydactyly, obesity, cognitive impairment, and atrioventricular septal defect) mutated in *IFT27/BBS19*. IFT27 is part of the Intraflagellar transport (IFT), a bidirectional mechanism allowing the protein motility within the cilia. Using whole exome sequencing, two compound heterozygous mutations were found in the proband (NM_006860.4:c.[104A > G];[349+1G > T], p.[Tyr35Cys];[?]) consistent with the expected autosomal recessive inheritance mode. These two mutations have already been reported but independently in other families and lacking either familial segregation or functional validation. This is the third report of *IFT27* mutations in BBS patients confirming *IFT27* as a BBS gene (*BBS19*). Mutations in IFT genes (*IFT27, IFT172* and *IFT74*) confirm the IFT-pathway as a pathomechanism for BBS.

## Introduction

Bardet-Biedl syndrome (BBS; OMIM 209900) is a recessive and genetically heterogeneous ciliopathy defined by the association of retinitis pigmentosa (RP), postaxial polydactyly, obesity, hypogonadism, kidney dysfunction and cognitive impairment. Mutations in 22 genes have been identified of which *WDPCP/BBS15, LZTFL1/BBS17*, *BBIP1/BBS18* *IFT27/BBS19*, *IFT172/BBS20, C8Orf37/BBS21*, and *IFT74/BBS22* only account for one or a few families (Khan et al., 2016). Among the last 4 genes identified, 3 (*IFT27/BBS19*, *IFT172/BBS20*, and the candidate locus *IFT74/BBS22*) are members of the intraflagellar transport machinery (IFT) delineating a possible novel mechanisms for the BBS (Aldahmesh et al., 2014; Bujakowska et al., 2015; Lindstrand et al., 2016). The IFT machinery is composed of 2 large protein complexes named IFT-A and IFT-B required, respectively, for retrograde and anterograde transport in the cilia. This machinery is part of an essential process for the assembly and the maintenance of the cilia (Taschner and Lorentzen, 2016). Most of the reported mutations in these genes, and in particular for *IFT172*, have been identified in patients with skeletal forms of ciliopathies including the Jeune asphyxiating thoracic dystrophy (OMIM 208500), Mainzer–Saldino syndrome (OMIM 266920), the Sensenbrenner syndrome (OMIM 218330).

In this study, we report and validate 2 compound heterozygous mutations in *IFT27* confirming the 19th BBS locus in a family with a clear BBS phenotype.

## Materials and Methods

### Subjects

Bardet-Biedl syndrome studies have been approved by our Institutional Review Board “Comité de Protection des Personnes” (EST IV, N°DC-20142222) and written informed consent for research and publication was obtained from each participant. We declare that the present research complies with the declaration of Helsinki.

### Whole-Exome Sequencing

Whole exome sequencing (WES) was performed by the IGBMC Microarray and Sequencing platform. Genomic DNA (2 μg) was sheared to obtain a mean fragment size of 150 bp using the Covaris E210 (KBioscience, Herts, United Kingdom) followed by library preparation using the Agilent SureSelect XT Human all exon V6 (Agilent Technologies, Santa Clara, CA, United States PN G7530-90000, protocol B4). Sequencing was performed on an Illumina HiSeq 4000 (Illumina, San Diego, CA, United States) to generate 100-bp paired-end reads following the manufacturer’s protocols.

### Bioinformatics Analysis

Image analysis and base calling were performed using CASAVA v1.8.2 (Illumina) and the 109,658,782 reads were mapped to the reference human genome (GRCh37/hg19) using BWA v0.7.5a (Li and Durbin, 2009) leading to 95.89% of the bases covered at least by 20x. GATK UG v3.2-2 was used to call SNV and indel variations (DePristo et al., 2011). Annotation and ranking of SNV and indel were performed by VaRank (Geoffroy et al., 2015) in combination with the Alamut Batch software (Interactive Biosoftware, Rouen, France). Very stringent filtering criteria were applied to filter out non-pathogenic variants (Supplementary Table S1): (i) variants represented with an allele frequency of more than 1% in public variation databases including the 1000Genomes (Genomes Project et al., 2015), the gnomAD database (Lek et al., 2016), the DGV database (MacDonald et al., 2014) or our internal exome database, (ii) variants in 5′ and 3′ UTR, downstream, upstream or intronic locations and synonymous without pathogenic prediction of local splice effect, (iii) variants not in the ciliary genes (Nevers et al., 2017). Structural variants were predicted using CANOES (Backenroth et al., 2014) and annotated by AnnotSV (Geoffroy et al., 2018). Our analysis was focused on compound heterozygous and homozygous variants consistent with a recessive mode of transmission. The *IFT27* nomenclature is based on the RefSeq accession number NM_006860.4 (O’Leary et al., 2016).

### RNA Extraction, cDNA Synthesis, and Sanger Sequencing

RNA was extracted from the patient’s blood using the PAXgene Blood RNA Kit (PreAnalytiX GmbH, Hombrechtikon, Switzerland). Reverse transcription was performed on 200 ng of RNA using the BioRad iScript cDNA^®^ Synthesis Kit (BioRad, Hercules, CA, United States). PCR was performed on the cDNA using the Mastercycler ep. Gradient S thermocycler (Eppendorf, Germany). Specific fragment analysis was done by cutting the agarose gel around the fragment of interest (e.g., F3) and eluted in Tris–EDTA buffer 10.1 overnight. The elution product (5 μL) was reamplified by PCR using the same conditions to obtain a unique fragment. Bidirectional sequencing of the purified PCR products was performed by the GATC Sequencing Facilities (Konstanz, Germany). Primers used are summarized in Supplementary Table S2.

## Results

The patient is the only child of an unrelated couple without personal or familial medical history. The patient was born at 39 weeks of amenorrhea with the following parameters: weight at 3210 g, height at 51 cm and head circumference at 35 cm. Mesoaxial polydactyly of the right hand with a Y-shaped metacarpian and syndactyly between the 5th and the 6th fingers and postaxial polydactyly of the right foot was noticed at birth. Partial atrioventricular septal defect was also diagnosed and operated at 5 weeks old. Mitral insufficiency persisted after operation and was operated at 2 years old. Renal ultrasound was normal. At birth, cerebral ultrasound showed isolated thin corpus callosum, not confirmed on cerebral MRI. Secondarily, the patient presented with delayed psychomotor development: he walked at 25-month-old and had delayed language (5 words at 2 years; 10 words at 3 years and sentences at 5 years). Audition was normal. The patient received specialized education. Progressively, he developed obesity: at 2 years old, 16.8 kg (+ 3SD) for 93 cm (+ 2SD) and normal head circumference (49 cm); at 3 years old, 22.9 kg (> +3SD) for 101.5 cm (+ 2SD). At 7 years old, his BMI was 25. Initially, ophthalmologic examination revealed myopia at 2 years old and alternate divergent strabismus at 3 years old. Myopia was scalable was noticed at 4 years old. A cone-rod dystrophy was diagnosed at 7 years old.

**FIGURE 1:**
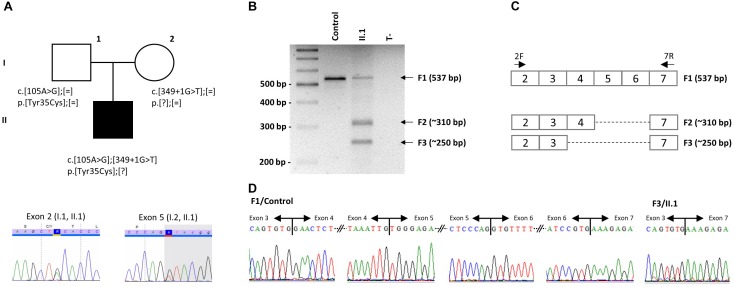
Identification of two mutations in *IFT27*. **(A)** Pedigree of the reported family with one affected individual and segregation analysis of the two IFT27 mutations. Example of Sanger sequencing profiles for the heterozygous individuals. **(B)** PCR amplification was performed on RNA extracted from blood of individual II.1 and a healthy unrelated control amplified between exon 2 and exon 7. **(C)** *IFT27* cDNA scheme representing the obtained fragments with size and expected composition. PCR primers are positioned. **(D)** Sanger sequencing of normal F1 in a healthy unrelated control (left side showing each exon boundaries from exon 3 to 7) and cut and eluted F3 band in individual II.1 demonstrating the absence of exons 4 to 6 (right side).

**FIGURE 2:**
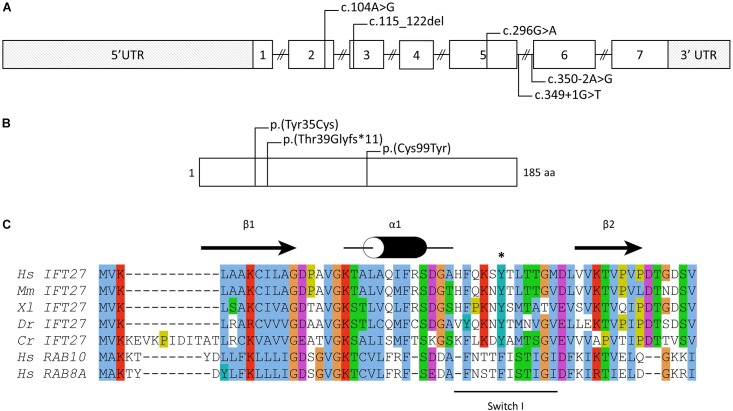
IFT27 gene and protein. **(A)** Schematic of the *IFT27* locus and **(B)** IFT27 protein and the position and/or nature of known mutations. Nomenclature is given according to the following RefSeq identifiers: NM_006860.4 and NP_006851.1. **(C)** Multiple sequence alignment of IFT27 proteins from different species and of the human RAB8a and RAB10 proteins. The secondary structures derived from the IFT27 structure are shown in black above the sequences (Bhogaraju et al., 2011). The mutation Tyr35 is highlighted using a black “^∗^” and the Switch I region is shown using a black line below the sequences. Residues are presented and colored according to the Jalview program (Waterhouse et al., 2009). Hs, *Homo sapiens*; Mm, *Mus musculus*; Xl, *Xenopus laevis*; Dr, *Danio rerio*; Cr, *Chlamydomonas reinhardtii*.

Several diagnostic hypotheses led to the non-conclusive exploration of *GLI3* (Pallister-Hall), *OFD1* (Orofaciodigital syndrome I) and the known BBS genes at that time (*BBS1-BBS18*) using Sanger and targeted exome sequencing (Muller et al., 2010; Redin et al., 2012). This also included array-CGH screening. Finally, WES was performed on the index case and led to the identification of 2 already reported variations in the *IFT27/BBS19* gene co-segregating in the family (Figure 1A). The first variation, c.104A > G (p.Tyr35Cys), is affecting a highly conserved position in the protein localized in the switch I region of this Rab-like GTPase (Figure 2A,B). The variation is absent from the variation databases (i.e., 1000 genomes and gnomAD) and predicted as affecting the protein function (Supplementary Table S3). Given its relatively close position toward the end of exon 2 (Figure 2A), a possible splice effect was assessed on the patient’s RNA extracted from blood and failed to reveal any alteration (Supplementary Figure S1).

Given its localization, the second variation, c.349 + 1G > T, is predicted to abolish the donor splice site of exon 5 (Figure 2A and Supplementary Table S3). It is already described in gnomAD with 21 heterozygous alleles in the European (Non-Finnish) cohort leading to an allele frequency of 0.0085% compatible with the BBS rare condition. Analysis of the patient’s RNA extracted from blood revealed a mix of alternatively spliced isoforms not found in controls (Figure 1B–D). This includes removal of exons 5 + 6 or 4 + 5 + 6 that are predicted to cause an in frame deletion of a significant part of the protein (76 or 96aa out of 185aa).

## Discussion

Bardet-Biedl syndrome is an emblematic ciliopathy characterized by a clinical and genetic heterogeneity. Twenty-two BBS genes have been identified so far and mutations are found in about 80% of the known BBS patients (Redin et al., 2012; Khan et al., 2016). The last genes identified account only for a small fraction of the mutation load, emphasizing the importance to screen more families and increase our knowledge for these genes (e.g., type of mutations and clinical manifestations).

In our study, we identified two compound heterozygous mutations in *IFT27* (NM_006860.4: c.[104A > G];[349 + 1G > T]) by WES in a child presenting with the classical BBS signs. IFT27 is part of the RAb like GTPAse that is predicted to have low GTPAse activity. The gene is composed of 7 exons coding for a 186 aa protein.

Our first identified mutation, the c.104A > G is located in exon 2 leading to the drastic change of a tyrosine to a cysteine (p.Tyr35Cys). Localized in the connecting region between α-helix 1 and β-sheet 2 in the so called “switch I” region (Figure 2C), a highly flexible region upon nucleotide binding pocket not crystallized (Bhogaraju et al., 2011) with an important role in GDP/GTP exchange and with interacting partners (Pylypenko et al., 2018), the missense is highly susceptible to affect the function of IFT27. The second mutation, c.349 + 1G > T, located in the donor splice site of exon 5 was predicted to affect the splicing of *IFT27* that we have demonstrated with a skipping of several exons of the gene. This mutation has been already reported in a fetus with a severe phenotype but the effect could not be assessed at that time (Quelin et al., 2018).

Initially the first identified mutations in *IFT27* were reported in a consanguineous BBS family presenting with RP, obesity, polydactyly of all extremities, mild intellectual disability, renal failure and hypogenitalism. The patients carried a homozygous missense (c.296G > A, p.Cys99Tyr) predicted to affect the stability of the protein (Aldahmesh et al., 2014).

Very recently, another BBS case with RP (cone rod dystrophy), obesity, polydactyly, maturation and learning delay and chronic renal failure has been reported but they could not to assess the segregation and the effect on the protein (c.[104A>G(;)350-2A>G], p.[Tyr35(;)?]) (Sanchez-Navarro et al., 2018).

Lastly, 2 loss of function mutations (c.[115_122del];[349+1G > T], p.[Thr39Glyfs^∗^11];[?]) have been reported in a fetus presenting with a severe ciliopathy with short ribs polydactyly type II (SRPII or Majewski syndrome) and/or Pallister-Hall syndrome (Quelin et al., 2018). Interestingly, Pallister-Hall syndrome was also evocated for our patient given the association of cardiopathy and polydactyly with Y-Shaped metacarpian. The authors made the hypothesis that the loss of function mutations could explain the severe phenotype.

Comparing the reported patients including the fetus (Supplementary Table S4), all had postaxial polydactyly. All living patients had RP, learning difficulties and obesity. Moreover, one patient of the initial BBS family has deafness and hypogenitalism. The same family and the fetus have renal anomalies (renal hypoplasia to renal agenesis) contrasting with our patient who has no renal involvement. Finally, our patient and one patient of the initial family presented with a cardiopathy, and unexpectedly not found in the more severe fetal form. Interestingly some patients have unusual clinical presentation including mesoaxial polydactyly with Y-shaped metacarpian associated with postaxial polydactyly (our patient) or preaxial (fetus). The Y-shaped metacarpian has been already correlated with mutation in *BBS17/LZTFL1* (Schaefer et al., 2014) that is now extended to *IFT27*. One can notice also that 2 patients had cone-rod dystrophy (our patient and family 2), a rare condition already described in BBS (Scheidecker et al., 2015). These features show the clinical variability associated to *IFT27* mutations from severe lethal forms to classical BBS that might be revised when new patients will be described in the future.

Mutations in IFT genes were recently reported in several BBS patients (Bujakowska et al., 2015; Lindstrand et al., 2016; Schaefer et al., 2016). *IFT27, IFT74*, and *IFT172* encode components of the IFT-B complex required for anterograde transport of ciliary proteins (Taschner and Lorentzen, 2016). Mutations in IFT genes are implicated in different ciliopathies, principally in skeletal ciliopathies (Jeune asphyxiating thoracic dystrophy, Sensenbrenner syndrome and Mainzer-Saldino syndrome) but also in isolated RP and Senior-Løken syndrome (associating RP to nephronopthisis). This report confirms the IFT-pathway as a new pathomechanism for BBS.

## Conclusion

We identified the third BBS family mutated in *IFT27.* We confirmed *IFT27* as the 19th BBS gene delineating the typical and complete BBS phenotype for those rarely mutated patients and the implication of IFT-pathway in the BBS pathogenesis. This report illustrated the usefulness of WES sequencing to identify mutations in highly heterogeneous genetic disorders. Finally, our observation is a good illustration of the clinical and molecular continuum between the different ciliopathies.

## Data Availability

All variants have been submitted to ClinVar using the following range of accessions numbers SCV000839871 and SCV000839872 (https://www.ncbi.nlm.nih.gov/clinvar/).

## Author Contributions

HD and JM conceived the study. CM-D, JG, and MH-E collected the clinical information. CD, CS, ES, and LM analyzed the data. VG conducted the bioinformatics analysis. ES and JM drafted the manuscript. HD and JM supervised the entire study. All the authors approved the final version of manuscript.

## Conflict of Interest Statement

The authors declare that the research was conducted in the absence of any commercial or financial relationships that could be construed as a potential conflict of interest.
