# Supplementary material for: Identification and Characterization of Known Biallelic Mutations in the IFT27 (BBS19) Gene in a Novel Family With Bardet-Biedl Syndrome
Source: Front Genet. 2019 Jan 30;10:21. doi: 10.3389/fgene.2019.00021 (PMC6363664; doi:10.3389/fgene.2019.00021)
Supplement: Supplementary file 1 [file Data_Sheet_1.docx]

**Supplementary Data**

|  | SNV | indel | SV |
| --- | --- | --- | --- |
| Total number of variants | 92351 | 14676 | 12 |
| After exclusion of variants with an allele frequency >1% (gnomAD, 1000G, internal exome database or DGV) (1-3) | 1847 | 284 | 6 |
| After exclusion of SNV/indel found at the homozygous state in gnomAD and in our internal exome database | 1531 | 255 | 6 |
| After exclusion of SNV/indel in 5' and 3'UTR, downstream, upstream, intron and synonymous locations without local splice effect prediction | 559 | 84 | 6 |
| After exclusion of missense without SIFT, PPH2 or PhastCons deleterious effect | 500 | 84 | 6 |
| After exclusion of variants absent from ciliary gene list (4) | 84 | 8 | 0 |
| After selection of variants consistent with recessive transmission (compound heterozygous, homozygous variants) | 17 compounds heterozygous in: *ACADM, BOLA3, DAW1, DIMT1, DNAH8, EPB41L1, IFT27, KDM4B, KNTC1, LRRC56, LRTOMT, PPP3CC, SEC63, SMARCA5, SYNE2*, *TCTN1* and *USP25* | | |

Supplementary Table 1. Summary of the whole exome sequencing results. SNV: single nucleotide variation, indel: gain or loss of up to 50 nucleotides at a single locus, SV: structural variation. Exclusion of SV with a DGV frequency >1% is done only with studies of more than 1000 individuals.

| Application | Exon | Forward (5'-3') | Reverse (5'-3') | Size |
| --- | --- | --- | --- | --- |
| DNA | 2 | catagggcgtgtttcagtggaaga | ccaagttgcttgtggccattctg | 600 bp |
|  | 5 | tctaccgagttgggaggtttcaca | tccctggggttagctttctggaat | 710 bp |
| cDNA | 2 to 7 | CAGATCTTCCGCAGTGATGG | TCTGTCTTCTCCGGTTGTGC | 537 bp |
|  | 1 to 3_4 | CCATGGTGAAGCTGGCAG | GTTCCACACTGTCTCCCGT | 177 bp |

Supplementary Table 2. List of primers used in this study (*IFT27*: NM_006860.4).

|  | Exon | MaxEntScan | NNSplice | SSF | Align-GVGD | PolyPhen-2 | SIFT |
| --- | --- | --- | --- | --- | --- | --- | --- |
| c.104A>G, p.Y35C | 2 |  |  |  | Deleterious (Class 65) | Probably damaging  (1.000) | Affect protein function (0.00) |
| c.349+1 | 5 | -100% | -100% | -100% |  |  |  |

Supplementary Table 3. Prediction of the effect of the two variations on the protein function. Variant effect on the nearest splice site was predicted using MaxEntScan (5), NNSplice (6) and Splice Site Finder (7) by calculating score change between the wild type and the mutated sequences expressed as a percent differences. Missense effect was assessed by Align-GVGD (8), PolyPhen-2 (9) and SIFT (10).

|  | Ethnicity | Consanguinity | Segregation | Genotype | RP | Obesity | Polydactyly | CI | Renal  defect | Genital  defect | Anosmia | Others | Reference |
| --- | --- | --- | --- | --- | --- | --- | --- | --- | --- | --- | --- | --- | --- |
| Family 1 | Saudi Arabia | Y | Y | c.[296G>A];[296G>A]  p.[(Cys99Tyr)];[(Cys99Tyr)] | Y | Y | Y | Y | Y | Y | Y | Deafness, atopy, congenital heart disease, fatty liver | (11) |
|  | Saudi Arabia |  | Y | c.[296G>A];[296G>A]  p.[(Cys99Tyr)];[(Cys99Tyr)] | Y | Y | Y | Y | N | N | Y | Atopy, fatty liver | (11) |
| Family 2 |  | N | N | c.[104A>G(;)350-2A>G]  p.[Tyr35Cys(;)?] | Y^b^ | Y | Y | Y | Y | N | N |  | (12) |
| Family 3^a^ |  | N | Y | c.[115_122del];[349+1G>T]  p. [Thr39Glyfs*11];[?] |  |  | Y^c^ |  | Y | Y |  | Short ribs, imperforate anus, laryngeal anomalies | (13) |
| Family 4 | Caucasian | N | Y | c.[104A>G];[349+1G>T]  p.[(Tyr35Cys)];[?] | Y^b^ | Y | Y^c^ | Y | N | N | N | Atrioventricular septal defect, Y-shaped metacarpian | This study |

**Supplementary Table 4.** Genotype and phenotype summary of the reported *IFT27* patients. RP: retinitis pigmentosa, CI: cognitive impairment, ^a^this case is a fetus, ^b^cone-rod dystrophy, ^c^mesoaxial.


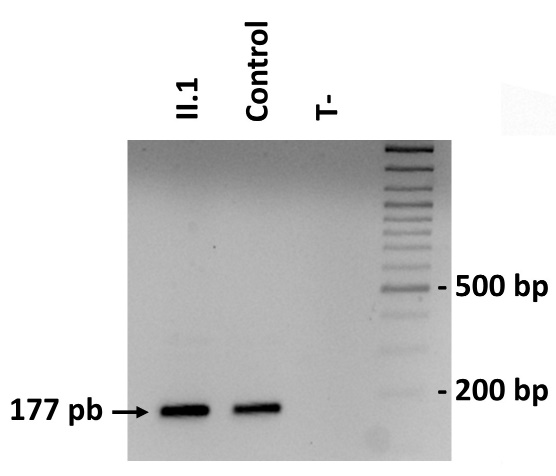


**Supplementary Figure 1.** Cys35Tyr possible splice effect assessment. PCR amplification was performed on RNA extracted from blood of individual II.1 and a healthy unrelated control amplified between exon 1 and exon 3/4.

**Supplementary References**

1. Genomes Project C, Auton A, Brooks LD, Durbin RM, Garrison EP, Kang HM, et al. A global reference for human genetic variation. *Nature* (2015) 526(7571):68-74. doi: 10.1038/nature15393. PubMed PMID: 26432245; PubMed Central PMCID: PMCPMC4750478.

2. Lek M, Karczewski KJ, Minikel EV, Samocha KE, Banks E, Fennell T, et al. Analysis of protein-coding genetic variation in 60,706 humans. *Nature* (2016) 536(7616):285-91. doi: 10.1038/nature19057. PubMed PMID: 27535533; PubMed Central PMCID: PMCPMC5018207.

3. MacDonald JR, Ziman R, Yuen RK, Feuk L, Scherer SW. The Database of Genomic Variants: a curated collection of structural variation in the human genome. *Nucleic Acids Res* (2014) 42(Database issue):D986-92. doi: 10.1093/nar/gkt958. PubMed PMID: 24174537; PubMed Central PMCID: PMC3965079.

4. Nevers Y, Prasad MK, Poidevin L, Chennen K, Allot A, Kress A, et al. Insights into Ciliary Genes and Evolution from Multi-Level Phylogenetic Profiling. *Mol Biol Evol* (2017) 34(8):2016-34. Epub 2017/05/02. doi: 10.1093/molbev/msx146. PubMed PMID: 28460059; PubMed Central PMCID: PMCPMC5850483.

5. Yeo G, Burge CB. Maximum entropy modeling of short sequence motifs with applications to RNA splicing signals. *Journal of computational biology : a journal of computational molecular cell biology* (2004) 11(2-3):377-94. doi: 10.1089/1066527041410418. PubMed PMID: 15285897.

6. Reese MG, Eeckman FH, Kulp D, Haussler D. Improved splice site detection in Genie. *Journal of computational biology : a journal of computational molecular cell biology* (1997) 4(3):311-23. Epub 1997/10/01. PubMed PMID: 9278062.

7. Shapiro MB, Senapathy P. RNA splice junctions of different classes of eukaryotes: sequence statistics and functional implications in gene expression. *Nucleic Acids Res* (1987) 15(17):7155-74. PubMed PMID: 3658675; PubMed Central PMCID: PMC306199.

8. Tavtigian SV, Deffenbaugh AM, Yin L, Judkins T, Scholl T, Samollow PB, et al. Comprehensive statistical study of 452 BRCA1 missense substitutions with classification of eight recurrent substitutions as neutral. *J Med Genet* (2006) 43(4):295-305. Epub 2005/07/15. doi: 10.1136/jmg.2005.033878. PubMed PMID: 16014699; PubMed Central PMCID: PMCPMC2563222.

9. Adzhubei IA, Schmidt S, Peshkin L, Ramensky VE, Gerasimova A, Bork P, et al. A method and server for predicting damaging missense mutations. *Nat Methods* (2010) 7(4):248-9. Epub 2010/04/01. doi: 10.1038/nmeth0410-248. PubMed PMID: 20354512; PubMed Central PMCID: PMCPMC2855889.

10. Kumar P, Henikoff S, Ng PC. Predicting the effects of coding non-synonymous variants on protein function using the SIFT algorithm. *Nat Protoc* (2009) 4(7):1073-81. Epub 2009/06/30. doi: 10.1038/nprot.2009.86. PubMed PMID: 19561590.

11. Aldahmesh MA, Li Y, Alhashem A, Anazi S, Alkuraya H, Hashem M, et al. IFT27, encoding a small GTPase component of IFT particles, is mutated in a consanguineous family with Bardet-Biedl syndrome. *Hum Mol Genet* (2014) 23(12):3307-15. Epub 2014/02/04. doi: 10.1093/hmg/ddu044. PubMed PMID: 24488770; PubMed Central PMCID: PMCPMC4047285.

12. Sanchez-Navarro I, L RJdS, Blanco-Kelly F, Zurita O, Sanchez-Bolivar N, Villaverde C, et al. Combining targeted panel-based resequencing and copy-number variation analysis for the diagnosis of inherited syndromic retinopathies and associated ciliopathies. *Sci Rep* (2018) 8(1):5285. Epub 2018/03/29. doi: 10.1038/s41598-018-23520-1. PubMed PMID: 29588463; PubMed Central PMCID: PMCPMC5869593.

13. Quelin C, Loget P, Boutaud L, Elkhartoufi N, Milon J, Odent S, et al. Loss of function IFT27 variants associated with an unclassified lethal fetal ciliopathy with renal agenesis. *Am J Med Genet A* (2018). Epub 2018/04/29. doi: 10.1002/ajmg.a.38685. PubMed PMID: 29704304.
